# NsrR: a key regulator circumventing *Salmonella enterica* serovar Typhimurium oxidative and nitrosative stress *in vitro* and in IFN-*γ*-stimulated J774.2 macrophages

**DOI:** 10.1099/mic.0.2006/003731-0

**Published:** 2007-06

**Authors:** Nicola J. Gilberthorpe, Margaret E. Lee, Tania M. Stevanin, Robert C. Read, Robert K. Poole

**Affiliations:** 1 Department of Molecular Biology and Biotechnology, University of Sheffield, Sheffield S10 2TN, UK; 2 Academic Unit of Infection and Immunity, University of Sheffield Medical School, Royal Hallamshire Hospital, Sheffield S10 2RX, UK

## Abstract

Over the past decade, the flavohaemoglobin Hmp has emerged as the most significant nitric oxide (NO)-detoxifying protein in many diverse micro-organisms, particularly pathogenic bacteria. Its expression in enterobacteria is dramatically increased on exposure to NO and other agents of nitrosative stress as a result of transcriptional regulation of *hmp* gene expression, mediated by (at least) four regulators. One such regulator, NsrR, has recently been shown to be responsible for repression of *hmp* transcription in the absence of NO in *Escherichia coli* and *Salmonella*, but the roles of other members of this regulon in *Salmonella*, particularly in surviving nitrosative stresses *in vitro* and *in vivo*, have not been elucidated. This paper demonstrates that an *nsrR* mutant of *Salmonella enterica* Serovar Typhimurium expresses high levels of Hmp both aerobically and anaerobically, exceeding those that can be elicited *in vitro* by supplementing media with *S*-nitrosoglutathione (GSNO). Elevated transcription of *ytfE*, *ygbA*, *hcp* and *hcp* is also observed, but no evidence was obtained for *tehAB* upregulation. The hyper-resistance to GSNO of an *nsrR* mutant is attributable solely to Hmp, since an *nsrR hmp* double mutant has a wild-type phenotype. However, overexpression of NsrR-regulated genes other than *hmp* confers some resistance of respiratory oxygen consumption to NO. The ability to enhance, by mutating NsrR, Hmp levels without recourse to exposure to nitrosative stress was used to test the hypothesis that control of Hmp levels is required to avoid oxidative stress, Hmp being a potent generator of superoxide. Within IFN-*γ*-stimulated J774.2 macrophages, in which high levels of nitrite accumulated (indicative of NO production) an *hmp* mutant was severely compromised in survival. Surprisingly, under these conditions, an *nsrR* mutant (as well as an *nsrR hmp* double mutant) was also disadvantaged relative to the wild-type bacteria, attributable to the combined oxidative effect of the macrophage oxidative burst and Hmp-generated superoxide. This explanation is supported by the sensitivity *in vitro* of an *nsrR* mutant to superoxide and peroxide. Fur has recently been confirmed as a weak repressor of *hmp* transcription, and a *fur* mutant was also compromised for survival within macrophages even in the absence of elevated NO levels in non-stimulated macrophages. The results indicate the critical role of Hmp in protection of *Salmonella* from nitrosative stress within and outside macrophages, but also the key role of transcriptional regulation in tuning Hmp levels to prevent exacerbation of the oxidative stress encountered in macrophages.

## INTRODUCTION

The ability of *Salmonella enterica* serovar Typhimurium (*S. typhimurium*) to survive and proliferate within innate immune cells such as macrophages is central to its capacity to cause disease. The *S. typhimurium* genome encodes many mechanisms that allow resistance to the stressful environment encountered within the macrophage. Such stresses include the production by NAD(P)H oxidase (Phox) of superoxide anion (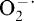) and other reactive oxygen species (ROS), and by inducible nitric oxide (NO) synthase (iNOS) of NO and other reactive nitrogen species (RNS) (Vazquez-Torres & Fang, 2001a; Fang, 2004).

The production of ROS, particularly 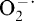, in the univalent reduction of O_2_ by Phox is often referred to as the oxidative burst, and is thought to be activated around 1 h after infection (Tsolis *et al.*, 1995). On activation of the phagocyte by uptake of *Salmonella*, the membrane and cytosolic components assemble in the cytoplasmic membrane in a process involving phosphorylation and cytoskeletal elements (Vazquez-Torres & Fang, 2001a; Forman & Torres, 2002). The 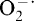 produced damages iron–sulfur [Fe–S] clusters and other targets (Imlay, 2002), whilst the hydrogen peroxide (H_2_O_2_) arising from 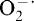 dismutation oxidizes protein thiols and [Fe–S] clusters and can create carbonyl and methionine sulfoxide adducts in proteins, thiols and membranes (Imlay, 2002). Furthermore, Fe(II) (formed, for example, by the reduction of Fe(III) by 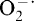 anion) reacts with H_2_O_2_ to generate the hydroxyl radical, which is a sufficiently powerful oxidant to react with virtually all organic molecules, including nucleic acids. To protect against oxidative stress, *S. typhimurium* possesses several antioxidant mechanisms (Farr & Kogoma, 1991; Vazquez-Torres & Fang, 2001b) that include not only superoxide dismutases (SODs) and hydroperoxidases, but also a type III secretory system that interferes with the trafficking of vesicles containing Phox to the phagosome (Fang & Vazquez-Torres, 2002).

The production of RNS by macrophages is mediated by iNOS (Vazquez-Torres *et al.*, 2000). Nitric oxide synthases (NOS) produce NO by the oxidation of a nitrogen atom of l-arginine to NO, using O_2_ and reducing equivalents provided by NAD(P)H as substrates. iNOS, the form of NOS most associated with antimicrobial activity, is primarily regulated at the transcriptional level and can be stimulated by the presence of microbial products and cytokines such as TNF*α*, IL-1 and IFN-*γ* (Vazquez-Torres *et al.*, 2000; Kalupahana *et al.*, 2005). Work by Chakravortty *et al.* (2002) demonstrated that the *Salmonella* pathogenicity island 2 (SPI-2) secretion system is able to interfere with the localization of iNOS and therefore aid avoidance of RNS. The extensive increase in production of RNS by macrophages following infection, often called the nitrosative burst, is thought to begin at around 8 h after infection in mouse macrophages (Eriksson *et al.*, 2003), but may begin much earlier in human macrophages (Stevanin *et al.*, 2002).

The protein considered most important in the detoxification of NO by *S. typhimurium* in aerobic conditions is the flavohaemoglobin Hmp. Flavohaemoglobins are the best-characterized class of microbial globin. They comprise two domains, a globin domain with a non-covalently bound haem B and a flavin domain with recognizable binding sites for FAD and NAD(P)H (Wu *et al.*, 2003). Hmp was first identified in *Escherichia coli* (Vasudevan *et al.*, 1991) and now has a clearly defined role in NO biology in that organism: its synthesis is markedly upregulated by NO (Poole *et al.*, 1996), and *hmp* knockout mutants of *E. coli* and *S. typhimurium* are severely compromised for survival in the presence of NO *in vitro* (Crawford & Goldberg, 1998b; Membrillo-Hernandez *et al.*, 1999). *Salmonella* Hmp has also been implicated in response to NO in human macrophages (Stevanin *et al.*, 2002). In the presence of molecular O_2_, Hmp catalyses an oxygenase (Gardner *et al.*, 1998) or denitrosylase (Hausladen *et al.*, 1998) reaction in which NO is stoichiometrically converted to 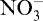 (Gardner *et al.*, 1998), which is relatively innocuous. Extensive studies of the purified protein (e.g. Mills *et al.*, 2001) have revealed some details of the reaction mechanism.

Regulation of Hmp levels in response to NO and related species in *E. coli* is complex. Control occurs predominantly at the transcriptional level and was shown in earlier studies to involve Fnr (Poole *et al.*, 1996; Cruz-Ramos *et al.*, 2002) and MetR (Membrillo-Hernandez *et al.*, 1998). Recent computational and experimental studies have also implicated NsrR [product of the *yjeB* (*nsrR*) gene] in *hmp* regulation (Rodionov *et al.*, 2005; Bodenmiller & Spiro, 2006). NsrR is an NO-sensitive transcriptional regulator of *hmp* and other genes known to be involved in nitrosative stress tolerance. It is a member of the Rrf2 family of transcriptional regulators, which also includes the IscR regulator that is involved in regulation of genes involved in [Fe–S] cluster biogenesis (Schwartz *et al.*, 2001). Based on the similarity of NsrR to IscR, which contains an [Fe–S] cluster (Schwartz *et al.*, 2001), and other members of the Rrf2 family (discussed in Rodionov *et al.*, 2005), it has been suggested (Bodenmiller & Spiro, 2006) that NsrR contains an NO-sensitive [Fe–S] cluster. Very recently, a similar conclusion has been reached for NsrR in *Bacillus subtilis* (Nakano *et al.*, 2006) and a role for NsrR in *S. typhimurium hmp* regulation has been proposed (Bang *et al.*, 2006). In *Salmonella*, the response of *hmp* transcription to 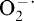 (generated by addition to cells of paraquat) is mediated by a further regulator, RamA (Hernandez-Urzua *et al.*, 2007). There has been some confusion over the possible role of the ferric uptake regulation (Fur) protein in *hmp* regulation. Crawford & Goldberg (1998a) proposed that the iron-responsive regulator, Fur, represses *hmp* transcription and that this repression is lifted by NO on inactivation of Fur. Although these results have been retracted (Crawford & Goldberg, 2006), and others have suggested that Fur is not involved in *Salmonella hmp* expression (Bang *et al.*, 2006), other promoters including *hmp* are controlled by nitrosylation of the Fur iron (D'Autreaux *et al.*, 2002). Furthermore, we have recently published evidence based on newly constructed *hmp–lacZ* fusions and immunoblotting that Fur is a repressor of *hmp* transcription in both *E. coli* and *Salmonella*, albeit a weak one (Hernandez-Urzua *et al.*, 2007). Given the global importance of Fur in intracellular iron management, a *fur* mutant of *S. typhimurium* might be compromised in its ability to resist killing within macrophages; conversely the modest upregulation of Hmp in the absence of Fur might confer a selective advantage.

The main aim of this study was to investigate the roles of members of the NsrR regulon, including Hmp, in surviving nitrosative stresses *in vitro* and *in vivo*. The ability to enhance, by mutating NsrR, Hmp intrabacterial levels without recourse to exposure to nitrosative stress also allowed us to test the hypothesis that NsrR plays a key role in tuning Hmp levels, since we have previously demonstrated that, *in vitro*, Hmp is a potent generator of the products of partial oxygen reduction (Membrillo-Hernandez *et al.*, 1996; Wu *et al.*, 2004). In this paper, we report roles *in vitro* and *in vivo* for components of the NsrR regulon in *S. typhimurium* determined by studying the phenotypes of *nsrR* and *hmp* mutants, and of an *nsrR hmp* double mutant in which all regulon components other than Hmp are upregulated. We also re-examine the role of Fur and demonstrate that a *fur* mutant is compromised for survival within macrophages even in the absence of elevated NO levels in non-stimulated macrophages.

## METHODS

### Bacterial strains, media and growth conditions.

All studies were performed using wild-type *Salmonella enterica* Serovar Typhimurium ATCC 14028s or SL1344 and their isogenic derivatives (Table 1). Bacteria were cultured at 37 °C, with cultures shaking at 220 r.p.m., in LB broth (Sambrook *et al.*, 1989) containing kanamycin (final concentration, 50 μg ml^−1^) or chloramphenicol (Cm) (final concentration, 25 μg ml^−1^) where appropriate. Media were inoculated at 1 % (v/v) with an overnight culture unless stated otherwise. For assessing the sensitivity of strains to methyl viologen (paraquat), H_2_O_2_ (both from Sigma) and *S*-nitrosoglutathione (GSNO), antibiotics were omitted from media. For assessing viability, cultures were spotted on nutrient agar (Sigma) containing the appropriate antibiotics. GSNO was synthesized according to the method of Hart (1985) and kindly donated by M. N. Hughes, University College London, UK. NO was synthesized according to the method in Poole *et al.* (1996).

To assess sensitivity to GSNO (3 mM) and paraquat (500 μM), growth curves were determined for cells grown in the absence or presence of the compounds in 10 ml LB in 250 ml flasks with side arms. Growth was measured by culture turbidity using a Klett–Summerson photoelectric colorimeter (Klett Manufacturing Co.) equipped with a no. 66 (red) filter. For H_2_O_2_ sensitivity assays, cultures were grown in 25 ml LB in 100 ml conical flasks for 3 h prior to addition of 5 mM H_2_O_2_. Sensitivity to H_2_O_2_ was determined by comparison of viable cell counts immediately prior to H_2_O_2_ addition with those at 10 and 25 min following addition. Viable counts were determined by conventional methods.

For Western blots, cells were grown as follows: anaerobic cultures were grown in six 8 ml glass tubes, filled to the brim with LB and sealed with Suba seals (Fisher); a glass ball in each tube facilitated resuspension of cells that had settled during static incubation. Aerobically grown cells were grown in six 10 ml LB batches in 250 ml flasks with side-arm for each strain. When the cultures reached mid-exponential phase (5 h anaerobic growth and 3 h aerobic growth, corresponding to Klett ∼40 and ∼80, respectively), 1 mM GSNO (final concentration) was added to three of the tubes/flasks for each strain. Anaerobic additions were made through the Suba seal using a Hamilton syringe. Cells were incubated for a further 2 h before being pooled and harvested by centrifugation at 5000 ***g*** for 10 min, 4 °C. Cells were stored as pellets at −20 °C.

### Western blotting.

Pelleted cells were washed in 1 ml 50 mM Tris/HCl (pH 7.5), centrifuged at 13 800 ***g*** for 3 min and resuspended in 1 ml 50 mM Tris/HCl (pH 7.5). Over ice, each suspension was sonicated for 3×30 s with 1 min rests, at an amplitude of 10 μm. The disrupted cell suspension was centrifuged at 13 800 ***g*** and then the supernatant fractions were assayed for protein with the Bio-Rad protein assay kit and BSA as the standard. A sample (10 μg protein) of each sample was subjected to SDS-PAGE. Anti-Hmp antibody (Stevanin *et al.*, 2000) was diluted 2000-fold for use with a 2000-fold dilution of peroxidase-conjugated monoclonal anti-rabbit immunoglobulin G (*γ*-chain specific, clone RG-96, A-1949; Sigma) as the secondary antibody. Western blots were carried out as described by Renart & Sandoval (1984); detection was done by using enhanced chemiluminescence (Amersham Biosciences).

### Mutagenesis.

The *λ* Red system was used to promote replacement (first described in *E. coli*; Murphy, 1998) of a large portion of the *nsrR* gene with a Cm resistance (*cat*) gene. The *cat* gene from pACYC184 was PCR-amplified with primers having 40 bp of 5′ and 3′ flanking homology to the *S. typhimurium nsrR* gene. The linear DNA fragment was electroporated into wild-type *S. typhimurium* carrying pTP223 and transformants selected on nutrient agar containing Cm (final concentration 25 μg ml^−1^). Putative mutants were picked the following day and verified by PCR amplification of the *nsrR* region. The mutation was transduced into a clean wild-type background using P22 (Maloy *et al.*, 1996), selecting for Cm^R^.

### Cloning.

The wild-type *nsrR* gene was amplified with primers engineered with terminal cut sites for *Eco*RI and *Bam*HI restriction enzymes (at the 5′ and 3′ ends respectively). The PCR product was digested with the enzymes overnight at room temperature. pBR322 was simultaneously digested with the same enzymes at 4 °C. The restricted fragment and plasmid were then ligated with T4 DNA ligase (Promega), overnight at 4 °C. Ligation mixture (10 ng DNA) was used to transform competent DH5*α* cells (Invitrogen) and transformants were selected on nutrient agar containing 200 μg ampicillin ml^−1^. The recombinant plasmid was then reisolated using the Qiagen Qiaquick miniprep spin kit, and used to electroporate the mutant strain.

### J774.2 macrophage culture and infection.

Macrophages for infection with *Salmonella* were cultured for 3 days in 24-well flat-bottom plates (2×10^5^ cells per well) in Dulbecco’s Modified Eagle’s medium (DMEM) (D5796, Sigma) supplemented with 10 % fetal calf serum (FCS), at 37 °C in a humidified atmosphere containing 95 % air/5 % CO_2_. Where indicated, DMEM was supplemented with murine IFN-*γ* (RD Systems; 1000 U ml^−1^, final concentration) approximately 9 h prior to infection. For fluorescence microscopy, macrophages were cultured on sterile glass coverslips (BDH). Prior to infection, 12 ml LB was inoculated at 2 % with an overnight culture and cells incubated at 37 °C, shaking at 220 r.p.m., for ∼1.5 h or to an OD_600_ of ∼0.4, measured using a Jenway 6100 spectrophotometer, in cuvettes with a pathlength of 1 cm. Immediately prior to infection, bacteria were harvested by centrifugation for 3 min at 13 800 ***g*** and washed in phosphate-buffered saline, pH 7.4 (PBS). Bacteria were finally resuspended in DMEM+10 % FCS to give the appropriate c.f.u. ml^−1^, as determined by conventional methods.

### Fluorescence microscopy.

Fluorescence microscopy was performed as described in Read *et al.* (1996). In brief, cells were fixed with 100 μl 2 % paraformaldehyde and stained using the nucleic acid stain 4,6-diamidino-2-phenylindole (DAPI), followed by a solution containing rabbit anti-*Salmonella* O antibody (Difco) diluted 1 : 50 in PBS. Cells were counterstained with fluorescein isothiocyanate (FITC)-conjugated goat anti-rabbit IgG antibody (Difco), diluted 1 : 20 in PBS. Coverslips were finally removed, dried, mounted in Vectashield (Molecular Probes), and viewed at a magnification of ×1000 in a DMRB 1000 fluorescence microscope (Leica). Internalization of bacteria was determined by subtracting the number of extracellular bacteria, identified by their co-localization with FITC-conjugated antibody, from the total number of bacteria exhibiting the DAPI stain.

### Assay of intracellular *Salmonella* viability.

Macrophages were infected with *Salmonella* at an m.o.i. of 11.25 or 7.5, and incubated for 30 min at 37 °C in a humidified atmosphere (95 % air/5 % CO_2_) to allow internalization. Following incubation, wells were washed twice with PBS. At the time of sampling, J774.2 cells were lysed with 250 μl of 2 % sterile saponin for 20 min at 37 °C. Lysates were collected and wells washed with PBS (750 μl) to achieve maximum recovery of bacteria. The first samples were taken 30 min after infection, including three uninfected wells, after which the DMEM in the remaining wells was replaced with fresh DMEM containing gentamicin (100 μg ml^−1^) and incubated for a further 30 min to kill extracellular bacteria. The minimum bactericidal concentration for gentamicin of each strain was determined by conventional methods and was shown to be identical for all strains used. Subsequently, the media containing gentamicin at 100 μg ml^−1^ were replaced with media containing gentamicin at 25 μg ml^−1^ and incubated until lysis with saponin as described above. The cells were lysed at 30 min after initial infection, prior to gentamicin treatment, and at 4, 15 and 21 h after initial infection. All samples were taken in triplicate. Bacterial viability was determined using standard dilution techniques. Supernatants removed at 4, 15 and 21 h were stored at −21 °C for later nitrite and nitrate analyses.

### Minimum bactericidal concentration test.

Cells were grown and washed exactly as they would be prior to macrophage infection, except that resuspension was in DMEM that contained various concentrations of gentamicin, up to 200 μg ml^−1^. Cells were incubated for 30 min before harvest, resuspended in 1 ml PBS and finally plated (10 μl spots) on nutrient agar and incubated overnight.

### Assays of nitrite and nitrate accumulation in tissue culture supernatants.

Macrophage production of NO was measured by assaying 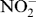 and 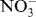 accumulation in culture supernatants as described in Stevanin *et al.* (2005) using a model 280i NO Analyser (Sievers).

### Quantitative real-time PCR (qRT-PCR).

RNA was extracted from 1 ml of a mid-exponential-phase aerobic culture of wild-type and *nsrR* strains using Qiagen RNAprotect and RNeasy kits as described by the manufacturer. Briefly, for each extraction, 1 ml culture was transferred into 2 ml RNAprotect reagent, followed by incubation at room temperature for 5 min. The samples were centrifuged at 6000 ***g*** for 10 min; the supernatant was discarded, and pellets frozen at −80 °C until use. The cell pellets were lysed after addition of 100 μl Tris-EDTA buffer containing lysozyme (Sigma) at a final concentration of 1 mg ml^−1^. RNA was purified as recommended by Qiagen and an on-column DNase digest was carried out using the RNase-free DNase set (Qiagen). RNA concentrations were determined spectrophotometrically using an Eppendorf Biophotometer. For cDNA synthesis, 4 μg RNA was added to 3 μl of a solution of random hexamer primers (Amersham Biosciences, 3 μg ml^−1^) and annealing was achieved by incubation at 65 °C for 10 min, 22 °C for 10 min and 2 min on ice. Reaction mixes (20 μl) containing 0.5 mM dATP, dTTP, dGTP and dCTP were incubated for 1 h at 42 °C with 200 units of Superscript II RNase-H Reverse Transcriptase (Invitrogen Superscript II kit). Following synthesis, cDNA was purified using a PCR purification kit (Qiagen) and eluted in 200 μl RNase-free water.

Gene-specific primers were designed to amplify 50- to 150- nucleotide fragments of target genes using PRIMER3 software (Rozen & Skaletsky, 2000). Each reaction was carried out in a total volume of 25 μl on a 96-well optical reaction plate (Applied Biosystems). Each well contained 0.5 μl 50× SYBR Green solution, 12.5 μl 2× Sensimix solution (Quantace), 3.25 pmol of each of the two primers and 5 μl cDNA sample. PCR amplification was carried out in an ABI 7700 thermocycler (PE Applied Biosystems) with the following thermal cycling conditions: 50 °C for 2 min; 95 °C for 10 min; 40 cycles of 95 °C for 15 s; 60 °C for 1 min. No-template reactions were included as negative controls. The data were analysed as described before (Flatley *et al.*, 2005).

### Reverse transcriptase PCR (RT-PCR).

cDNA was synthesized as described above; 1 μl of this was used as the template in a standard PCR reaction mixture using Accuzyme Polymerase (Bioline) to amplify.

### Determination of inhibition of respiration by NO.

The method was adapted from Stevanin *et al.* (2000). Cultures (25 ml) were grown for 5.5 h. Cells were harvested by centrifugation and resuspended in 2 ml PBS. The buffer was saturated with air in a Clark-type polarographic oxygen electrode system (Rank Bros), comprising a water-jacketed (37 °C) Perspex chamber stirred magnetically; the membrane-covered electrode was situated at the bottom of the chamber below the stirrer. Cell suspension (300 μl) was added, a close-fitting lid applied to the chamber, and an ISO-NOP NO sensor (2 mm diameter) (WPI) was inserted through a custom-made capillary hole in the lid. Oxygen levels in the chamber were allowed to fall by 50–60 % through respiration before 100 μl of anoxic, NO-saturated solution was injected into the chamber using a Hamilton microsyringe. Oxygen consumption and NO levels were measured until the chamber contents became anaerobic.

### Statistical analysis.

Parametric data were analysed for significance using the *t*-test and data plotted at means with error bars representing standard deviations (sd) or standard errors (sem) where stated. Non-parametric data were analysed for significance using the Wilcoxon signed rank test and data plotted as medians with error bars representing the 25th and 75th percentiles. Statistical significance was established at a *P* value of <0.05.

## RESULTS

### A *S. typhimurium nsrR* mutant constitutively synthesizes Hmp, aerobically and anaerobically

**Fig. 1. f1:**
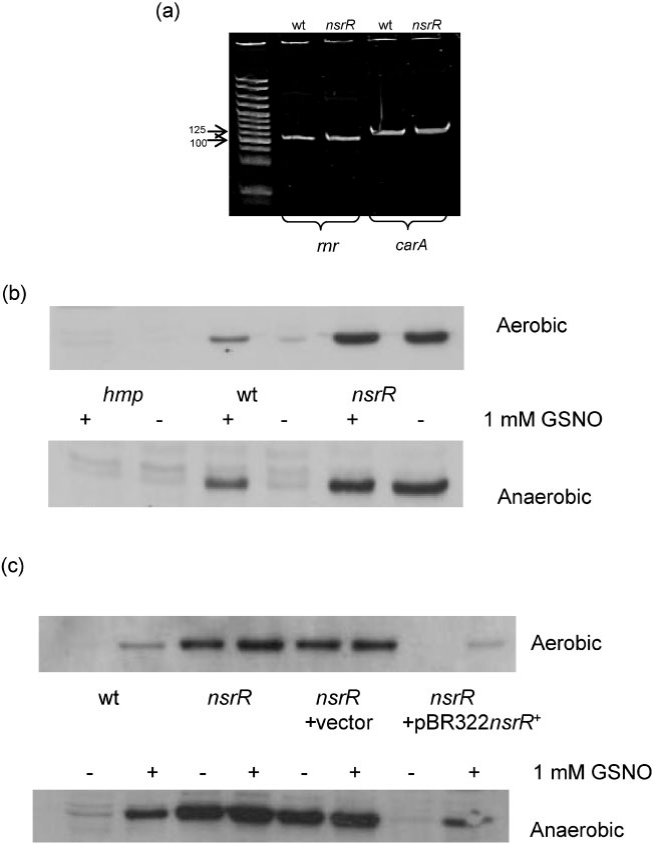
*rnr* transcript and Hmp protein levels in *Salmonella* mutants. (a) *rnr* expression levels in wild-type and *nsrR* mutant strains. Cells were grown aerobically in LB and harvested during mid-exponential phase. PCR was performed on cDNA from these cells. Panel (a) is representative of an experiment carried out in duplicate. (b) Expression of Hmp in wild-type (wt), *hmp* and *nsrR* strains grown aerobically or anaerobically. (c) Expression of Hmp in wild-type (wt), *nsrR*,* nsrR* carrying pBR322 and *nsrR* carrying pBR322*nsrR*^+^ grown aerobically or anaerobically. Cells were exposed where indicated (+) to 1 mM GSNO in LB for 2 h during exponential phase, prior to harvesting. Cell-free extracts were resolved by SDS-PAGE and Western blots performed using antibody raised to *E. coli* purified Hmp. Similar protein loadings (10 μg) were used for each gel lane.

An *nsrR* mutant was created by the insertion of a Cm resistance cassette in *S. typhimurium* using the *λ*-*red* recombination system (Poteete & Fenton, 1984). The mutation was transduced into a wild-type background using phage P22 and confirmed by PCR amplification of the *nsrR* region. Immediately downstream of *nsrR* is a gene encoding riboendonuclease R, *rnr*, formerly *vacB*. In *E. coli*, *nsrR* and *rnr* are co-transcribed (Cairrao *et al.*, 2003) and Rnr has been implicated in expression of virulence genes in *Shigella* and enteroinvasive *E. coli* (Tobe *et al.*, 1992). Since there is a possibility that this is also the case in *Salmonella*, a complementation test was conducted as follows. pBR322 was engineered to carry the wild-type allele of *nsrR* (pBR322*nsrR*^+^) and the plasmid used to transform *nsrR* mutant by electroporation. To verify that the *nsrR* phenotype described is due to mutation of *nsrR* and not due to polar effects on the *rnr* gene, PCR was carried out on *rnr* cDNA from both wild-type and *nsrR* strains. The expression of *rnr* and of the gene encoding carbomoyl phosphatase, *carA* (as control), were indistinguishable in the wild-type strain and *nsrR* mutant (Fig. 1a[Fig f1]).

Wild-type, *nsrR* and *hmp* strains were grown to mid-exponential phase, both aerobically and anaerobically, in LB medium in the presence or absence of 1 mM GSNO. Western blots were performed using rabbit anti-*E. coli*-Hmp polyclonal antibodies as a probe (Fig. 1b, c[Fig f1]) and clearly indicate the role of NsrR in the negative regulation of *hmp* in both aerobic and anaerobic conditions. An *hmp* mutant did not display a band corresponding to Hmp under any conditions. In the wild-type strain, a band corresponding to Hmp was detected only after cells were exposed to GSNO, whereas in the *nsrR* mutant strain a strong band corresponding to Hmp was observed in both the presence and absence of GSNO, illustrating loss of negative regulation in the mutant. Even in the presence of 1 mM GSNO, *hmp* was not maximally expressed in the wild-type strain, as demonstrated by comparison of the Hmp band intensity with that of the *nsrR* mutant. Our findings are in agreement with Rodionov *et al.* (2005), who used bioinformatics to predict that NsrR negatively regulates *hmp* and several other genes in a small regulon. Recently Bang *et al.* (2006) also identified NsrR as a regulator of Hmp in *S. typhimurium* using qRT-PCR. Western blots identical to those described above were also carried out on the *nsrR* strains carrying pBR322 and pBR322*nsrR*^+^ (Fig. 1c[Fig f1]) and show that Hmp is expressed as in the *nsrR* mutant in *nsrR* carrying pBR322 and as in the wild-type in *nsrR* carrying pBR322*nsrR*^+^; this expression of *nsrR*^+^ *in trans* restores its function as a repressor of *hmp* under non-nitrosating conditions.

### The *S. typhimurium* NsrR regulon contains at least four other genes

Rodionov *et al.* (2005) identified the following *S. typhimurium* genes as being potentially under the regulation of NsrR: the *hcp-hcr* (*nipAB*) operon, *hmp*, *ytfE* (*nipC*) and *tehB.* In addition they suggested that *E. coli ygbA* is also regulated by NsrR. We tested the influence of the *nsrR* mutation on the expression of these genes using qRT-PCR. In addition, we looked at the expression of *tehA*, which is located just upstream of *tehB*, and with which it may be co-transcribed. The upregulation of mRNA levels in the *nsrR* mutant strain in comparison to wild-type was as follows: *ytfE*, 544; *hcp*, 284; *ygbA*, 31.8; *hcr*, 4.1; *tehA*, 1.1; *tehB*, 1.1. Data presented here are representative of a single RT-PCR experiment using cDNA from wild-type and *nsrR* strains in technical triplicates. A biological repeat of this was carried out giving a similar trend in gene expression levels. *carA* was again used as the control gene.

The function of *ytfE* is unclear, although several global transcriptional analyses in *E. coli* have shown it to be highly induced under conditions of nitrosative stress (Mukhopadhyay *et al.*, 2004; Justino *et al.*, 2005; Pullan *et al.*, 2007). The *ygbA* gene also has no known function. *hcp* and *hcr* are both upregulated in our *nsrR* mutant. In *E. coli*, they are considered to be co-transcribed (van den Berg *et al.*, 2000), yet levels of *hcr* mRNA levels were only 4.1-fold greater in the mutant strain. This might be explained by differential stability of the mRNA transcript leading to faster degradation of the *hcr* portion of the transcript.

### The *hmp* gene is the NsrR regulon member conferring GSNO resistance

**Fig. 2. f2:**
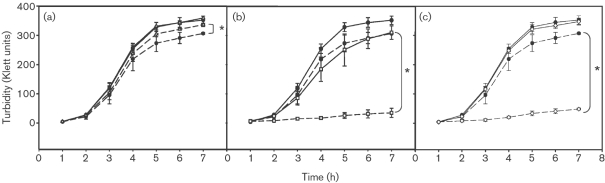
Hmp, but not other members of the NsrR regulon, confers growth tolerance to GSNO. Growth was in LB medium in the absence (solid lines) or presence (dashed lines) of 3 mM GSNO, measured turbidimetrically and shown as Klett units. (a) Comparison of wild-type (•) and *nsrR* (▵). (b) Comparison of wild-type (•) and *nsrR hmp* (□). (c) Comparison of wild-type (•) and *hmp* (◊). Data points are means±sd in three independent experiments. *, *P*=<0.05 at the 7 h data point using Student’s *t*-test.

On finding that *hmp* is constitutively expressed in an *nsrR* mutant (Fig. 1b[Fig f1]), we tested resistance of the mutant to nitrosative stress by growth in LB medium in the presence or absence of 3 mM GSNO. Fig. 2(a) shows that the aerobic growth characteristics of *nsrR* and wild-type strains are indistinguishable. In the presence of 3 mM GSNO, however, growth of the wild-type strain was more severely affected than that of the *nsrR* strain, presumably because enhanced expression of *hmp* in the *nsrR* strain affords more protection from GSNO. However, to establish if other members of the NsrR regulon protect against GSNO, an *nsrR hmp* double mutant was created by P22 transduction of the *hmp* mutation into the *nsrR* mutant, and the construct verified by Western blot analysis (not shown). Growth curves in the absence or presence of 3 mM GSNO (Fig. 2b[Fig f2]) show that removal of Hmp from the *nsrR* mutant abrogates the enhanced GSNO resistance of the *nsrR* mutant. Fig. 2(c) shows that a single *hmp* mutant has the same growth profile as the *nsrR hmp* double mutant in the presence of 3 mM GSNO. Mutants in *ytfE* and *hcp hcr* were also tested for their sensitivity to GSNO and were found to display growth profiles similar to that of wild-type (data not shown). Thus, no other members of the NsrR regulon are directly involved in conferring the ability to grow in the presence of GSNO.

### Overexpression of genes other than *hmp* can protect *S. typhimurium* respiration from inhibition by NO

**Fig. 3. f3:**
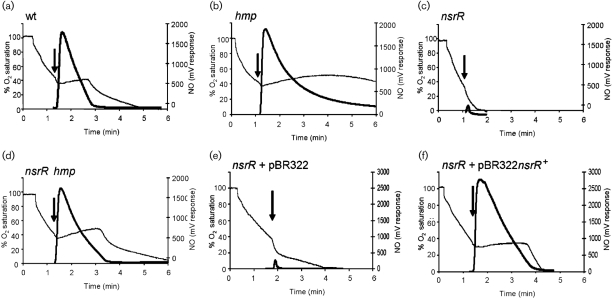
Hmp is the most significant member of the NsrR regulon conferring resistance of aerobic respiration to NO. Intact cells were allowed to respire in PBS until approximately 40 % of the dissolved oxygen remained, when 100 μl of an NO-saturated solution was injected. Fine line traces represent dissolved oxygen tension and thick lines represent NO. (a) Wild-type (wt); (b) *hmp* mutant; (c) *nsrR* mutant; (d) *nsrR hmp* mutant; (e) *nsrR* mutant carrying pBR322; (f) *nsrR* mutant carrying complementing plasmid. Data shown are representative of at least three independent experiments. Note that the recovery of respiration after NO inhibition in (a), (d) and (f) is spontaneous.

To test the effect of NO on respiration, mid-exponential-phase cells were allowed to respire aerobically in PBS until dissolved oxygen tension reached approximately 40 % of air saturation, at which point 100 μl NO-saturated solution was added to the cells. Fig. 3(a) shows that respiration of wild-type cells was totally but temporarily inhibited. NO addition was visualized by a rapid upward excursion of the NO electrode output while oxygen uptake was abruptly arrested for less than 2 min. After NO concentration fell, reaching negligible amounts approximately 1.5 min post-NO addition, oxygen uptake resumed at a rate similar to that before NO addition. The slight upwards deflection in the oxygen electrode traces seen after inhibition of respiration (and also in Fig. 3b and d[Fig f3]) presumably reflects polarographic drift or the back-diffusion of oxygen into the chamber through the capillary used for NO addition. A trace similar to that of wild-type can be seen in Fig. 3(f), where the *nsrR* mutant is complemented by pBR322*nsrR*^+^. A slightly prolonged inhibition of oxygen uptake can be seen in this trace, presumably attributed to multiple copies of the *nsrR* gene exerting higher repression on *hmp* than the single copy of *nsrR* in the wild-type genome. In the *hmp* strain (Fig. 3b[Fig f3]), although NO addition was evident by a similar spike to that seen in the wild-type experiment, the disappearance of NO was slower, and even after 6 min a significant amount of NO could be measured by the NO electrode. Oxygen uptake by the *hmp* mutant was severely affected by addition of NO and did not resume within 5 min of NO addition. Thus Hmp protects *S. typhimurium* from the effects of NO on aerobic respiration. Conversely, the *nsrR* mutant and the *nsrR* strain carrying pBR322, synthesizing high levels of Hmp, were unaffected by NO (Fig. 3c, e[Fig f3]); indeed, NO was consumed so quickly in each case that added NO was virtually undetectable by the electrode. Furthermore, directly following NO addition, a transient increase in oxygen uptake occurred, attributable to the Hmp-catalysed oxygenase mechanism (Gardner *et al.*, 1998). To assess the possible roles of other members of the NsrR regulon, the polarographic analyses were extended to the *nsrR hmp* mutant (Fig. 3d[Fig f3]). In this strain, despite the lack of Hmp-catalysed NO removal, NO was consumed more rapidly than for the *hmp* mutant (Fig. 3b[Fig f3]) and respiration resumed within 2 min of NO addition. We conclude that other gene(s) in the *nsrR* regulon, expressed to high levels in the *nsrR hmp* mutant, exert protective effects on respiration in the presence of NO. To try to establish which NsrR-regulated genes were responsible for protection in the *nsrR hmp* double mutants, mutants in *ytfE* and *hcp hcr* along with mutants in *nsrR ytfE* and *nsrR hcp hcr* were all tested for their ability to withstand NO inhibition of respiration. The *ytfE* and *hcp hcr* mutants showed profiles similar to that of wild-type (data not shown), and *nsrR ytfE* and *nsrR hcp hcr* mutants showed profiles similar to that of the *nsrR* mutant (data not shown). These results indicate that *ytfE* and *hcp hcr* do not have a role in protection of respiration from NO, when a functional *hmp* gene is still present. Several attempts were made throughout this study to construct an *ygbA* mutant to investigate its role in the above but were unsuccessful.

### *nsrR*, *hmp* and *fur* mutations attenuate survival in J774.2 macrophages

**Fig. 4. f4:**
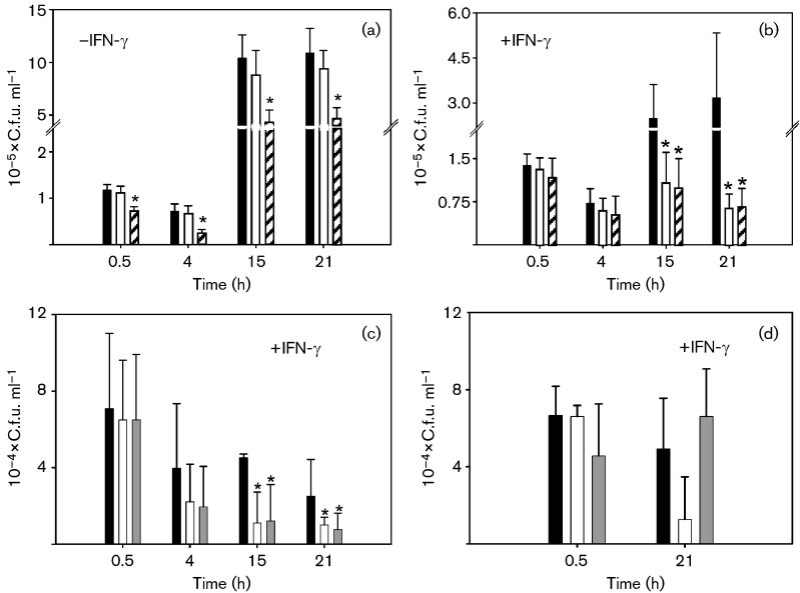
Intracellular survival of *Salmonella* is compromised in a *fur* mutant, but only in the presence of *γ*-IFN in an *hmp* mutant. Bacterial cells (∼2.5×10^6^) suspended in 250 μl DMEM+10 % FCS were used to infect ∼2×10^5^ J774.2 cells (a) and J774.2 cells stimulated with IFN-*γ* (b), giving an m.o.i. of ∼11. Bacterial cells (∼1.5×10^6^) similarly suspended were used to infect ∼2×10^5^ J774.2 cells (c), giving an m.o.i. of ∼7.5. The intracellular survival and proliferation was followed over time. At 0.5 h, cell counts include extracellular bacteria together with viable internalized bacteria. At subsequent time points, extracellular bacteria have been killed using gentamicin, leaving only viable intracellular bacteria. At each time point, counts for wild-type and for *hmp* and *fur* mutant strains are shown by the black, white and hatched bars, respectively in (a) and (b). In (c), at each time point, counts for wild-type and for *nsrR* and *nsrR hmp* mutant strains are shown by the black, white and shaded bars, respectively. In (d) counts for wild-type (wt), *nsrR* and *nsrR* carrying pBR322*nsrR* are shown by black, white and shaded bars, respectively. Results are medians±25th and 75th percentiles from between 6 and 12 separate experiments. *, *P*<0.05 using the Wilcoxon statistical analysis test.

In this study we used the mouse macrophage cell line J774.2. Prior to investigation of intracellular survival, each *Salmonella* strain was examined for the extent of association (defined here as the total number of bacteria either bound or internalized per macrophage), binding and internalization with J774.2, via fluorescence microscopy. After 30 min incubation, all bacterial strains associated similarly with the macrophages (data not shown) so that subsequent viable counts reflect intracellular survival and not differences in internalization. We also carried out gentamicin sensitivity tests to demonstrate that all strains were affected equally by the antibiotic used to kill extracellular bacteria following 30 min infection: all strains were equally sensitive to gentamicin (data not shown). Furthermore, the effects of bacterial infection on survival of J774.2 cells over 21 h were also examined; a trypan blue assay revealed insignificant effects on cytotoxicity (data not shown). Following infection (mean m.o.i. of 11.25), macrophages were lysed after 0.5, 4, 15 and 21 h and viable counts were carried out on the lysates to enumerate surviving intracellular bacteria. Fig. 4(a) shows that, in the absence of prior IFN-*γ* stimulation, there was no significant difference in the intracellular survival of wild-type and *hmp* mutant strains. However, mutation in the global iron response regulator, Fur, significantly affected the intracellular survival and proliferation of bacteria; resulting in significantly lower bacterial numbers being recovered, even after the initial 0.5 h infection period, indicating that the *fur* strain is unable to survive even the initial exposure to the intracellular macrophage environment. After 4 h infection, the number of recovered wild-type cells was over threefold higher than *fur* cells, and after 15 and 21 h wild-type counts were over double that of *fur* mutant counts (Fig. 4a[Fig f4]). These data suggest that the null *fur* mutant is severely attenuated in naïve macrophages, but that mutation in the NO-detoxifying globin gene, *hmp*, does not significantly affect the ability of *S. typhimurium* to survive under the same conditions. The data also suggest that *fur* is attenuated in these macrophages by an antimicrobial mechanism other than the nitrosative burst.

In subsequent experiments, macrophages were incubated with IFN-*γ* (1000 U ml^−1^) for approximately 9 h prior to infection. IFN-*γ* enhances a nitrosative stress response in macrophages upon infection with *S. typhimurium* (Kalupahana *et al.*, 2005). As expected (Liu *et al.*, 1999), the numbers of bacteria recovered from activated J774.2 cells were lower than those recovered from non-stimulated J774.2 cells for all three strains (Fig. 4b[Fig f4]), with wild-type numbers increasing only fivefold between 4 h and 21 h infection as compared to a 16-fold increase in non-activated cells (Fig. 4a[Fig f4]). The enhanced nitrosative stress associated with activation severely impairs survival and proliferation of the *hmp* mutant. The greatest differences between wild-type and both *hmp* and *fur* cell counts were seen at 15 and 21 h after infection, with numbers of wild-type reaching approximately two- and fourfold that of the mutant strains, respectively. These results clearly indicate the requirement for Hmp and Fur in maximal survival and proliferation in activated macrophages.

Since the *nsrR* mutant is hyper-resistant to GSNO (Fig. 2a[Fig f2]) and NO (Fig. 3c[Fig f3]), we hypothesized that this mutant may display additional resistance to killing by macrophages. Kim *et al.* (2003) reported that, when *hcp*, *hcr* and *ytfE* (*nipABC*) *Salmonella* mutants were used to infect mice, the ability of animals to clear the infection with these strains was diminished at low doses. We compared the survival of both the *nsrR* and *nsrR hmp* mutants to wild-type bacteria in IFN-*γ*-stimulated macrophages (Fig. 4c[Fig f4]) using a slightly lower m.o.i. of 7.5 to aid bacterial counting. Comparison of the wild-type strain in Fig. 4(b, c) indicates that a larger m.o.i. is required for proliferation of *S. typhimurium* in activated J774.2 cells over a 21 h period and that a decrease in m.o.i. (in this case of ∼33 %), does not correspond to an equal fall in c.f.u. ml^−1^ of wild-type *Salmonella* at 0.5 h (in this case it fell to ∼53 %), suggesting that J774.2 cells are more effective at limiting survival of *Salmonella* immediately after infection, and reducing subsequent proliferation, when the initial infection load is smaller. Our primary concern in this study was not to discern the correlation between infecting m.o.i. and survival of wild-type *Salmonella* but rather to assess whether there is a difference in the survival of wild-type and mutant strains of *Salmonella*. Surprisingly, we observed that both the *nsrR* and *nsrR hmp* mutants survived similarly intracellularly (Fig. 4c[Fig f4]), with bacterial counts for both strains being twofold lower than wild-type at 15 and 21 h lysis time points. A possible explanation of these data is that Hmp at high levels exerts toxic effects by the production of 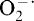 (Membrillo-Hernandez *et al.*, 1996; Wu *et al.*, 2004), so that the *nsrR* mutant, having elevated Hmp levels, is disadvantaged in macrophages due to exacerbated sensitivity to the oxidative response produced by the macrophages. To clarify that the *nsrR* mutation was not having polar effects on the downstream gene, *rnr*, which could have a role in virulence (Tobe *et al.*, 1992), intracellular survival of the *nsrR* mutant carrying pBR322*nsrR*^+^ was assessed in activated macrophages. Fig. 4(d) shows that inclusion of wild-type *nsrR* on a plasmid caused the intracellular survival of the *nsrR* mutant to return to the same level as the wild-type strain at 21 h.

### Nitrite and nitrate accumulation in tissue culture supernatants

**Fig. 5. f5:**
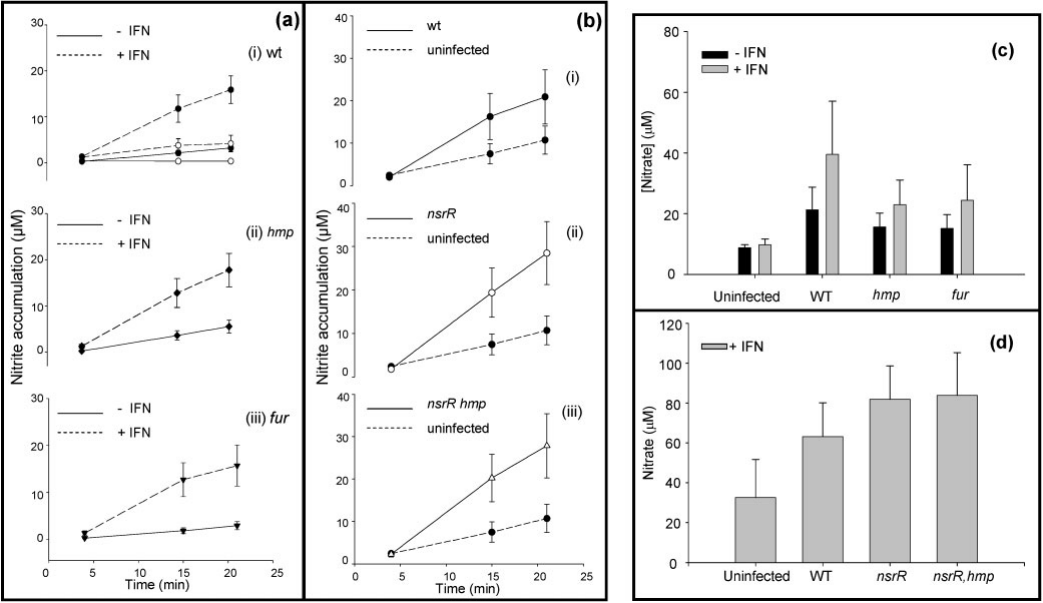
Nitrite and nitrate concentrations in supernatant fractions from intracellular killing assays. Tissue culture supernatants (DMEM+10 % FCS) were removed from macrophages immediately prior to lysis of macrophages. Panel (a) shows assay results for nitrite analysis in the absence and presence of IFN-*γ* for: (i) wild-type, (ii) *hmp* and (iii) *fur* bacteria. Uninfected J774.2 cells are shown by open circles in (i). Panel (b) shows results for nitrite analysis in the presence of IFN-*γ* for (i) wild-type, (ii) *nsrR* and (iii) *nsrR hmp* bacteria. Solid lines show the data for infected macrophages and dashed lines show the data for uninfected controls. Panel (c) shows nitrate analysis after 21 h for uninfected J774.2 cells and after infection with wild-type (wt), *hmp* and *fur* bacteria. Data are shown for the absence (black bars) and presence (grey bars) of IFN-*γ*. Panel (d) shows nitrate analysis after 21 h of uninfected J774.2 cells, wild-type (wt), *nsrR* and *nsrR hmp* bacteria. Concentrations of nitrite and nitrate represent the accumulation in fresh DMEM+10 % FCS media containing gentamicin (25 μg ml^−1^) that was applied to the macrophages immediately following 30 min incubation with DMEM+10 % FCS containing gentamicin (100 μg ml^−1^). Points and error bars represent means±sem of at least three experimental repeats.

We sought to verify the elevated production of NO in IFN-*γ*-stimulated J774.2 cells by assaying 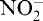 levels in tissue culture, as NO produced by iNOS is expected to be oxidized to 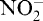 in the presence of oxygen (Ignarro *et al.*, 1993; Wink *et al.*, 1993; Kharitonov *et al.*, 1994). In the presence of Hmp, NO is detoxified to 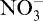 (Gardner *et al.*, 1998; Hausladen *et al.*, 1998). Supernatant fractions from non-stimulated macrophages accumulated very little 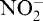 compared to their activated counterparts (Fig. 5a[Fig f5] ii). In non-activated, uninfected cells, 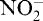 levels were ∼0.3 μM throughout the study, whereas in stimulated macrophages, 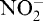 increased consistently throughout the experiment to ∼4 μM after 21 h, confirming activation of iNOS. 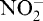 levels detected in non-activated J774.2 cells in the presence of wild-type (Fig. 5a[Fig f5] i) and *fur* (Fig. 5a[Fig f5] iii) strains were similar, with accumulations of around 3 μM after 21 h of infection whereas levels of 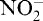 were slightly higher in *hmp*-infected J774.2 cells (5.5 μM) (Fig. 5a[Fig f5] ii). This could reflect the accumulation of NO in *hmp* infected cells and its oxidation to 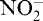, whereas in wild-type and *fur* strains, Hmp converts NO to 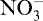. We tested this hypothesis by measuring the accumulation of 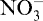 in tissue culture media over time. As with 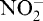, steady increases in 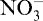 concentration were detected over the time-course of infection with all strains. Fig. 5(c) shows the total 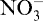 accumulated at 21 h. 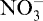 levels reached a mean of 8.7 μM in the supernatants of non-activated, uninfected cells after 21 h. 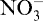 accumulation in wild-type-infected cells had a mean of 21.3 μM (Fig. 5c[Fig f5]). Both *hmp*- and *fur*-infected J774.2 tissue culture supernatants showed a similar accumulation of 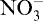, which was significantly lower than that of wild-type bacteria at around 15 μM. However, IFN-*γ*-activated J774.2 cells infected with *Salmonella* produced large amounts of 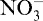 over 21 h (∼40 μM for the wild-type strain), compared to the non-stimulated, infected J774.2 cells. The 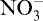 concentration in supernatants from stimulated macrophages was somewhat lower, at around 23 μM, for *hmp-* and *fur-*infected J774.2 cells (Fig. 5c[Fig f5]). The lower level of 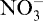 in the supernatant of *hmp*-infected cells could reflect an inability of the *hmp* mutant to detoxify NO to 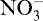; however, as the *fur* infected cells showed similar 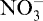 accumulation, it is probably more likely that differences in 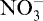 accumulation seen in both non-activated and activated tissue culture supernatants could be explained by the fact that iNOS activation may be infection-load dependent (Witthoft *et al.*, 1998).

Tissue culture supernatants were also collected from experiments where wild-type, *nsrR* and *nsrR hmp* strains were used to infect IFN-*γ*-stimulated macrophages and used to assay 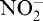
and 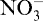 accumulations (Fig. 5b and d[Fig f5], respectively). Although *nsrR* mutant bacteria have elevated levels of Hmp relative to wild-type, no significant differences in 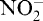 or 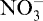 levels were detected, perhaps as a result of the loss of bacterial viability (see Fig. 4c[Fig f4]) and therefore lower activity levels of iNOS (Witthoft *et al.*, 1998). Macrophages infected with the *nsrR hmp* double mutant also showed no significant difference from the wild-type in 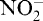 and 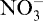 accumulation (Fig. 5b, d[Fig f5]), perhaps due to the lack of significant NO-metabolizing activity by the strain and the poor viability of such bacteria (Fig. 4c[Fig f4]).

### An *nsrR* mutation enhances sensitivity to oxidative stress, even in the absence of Hmp

**Fig. 6. f6:**
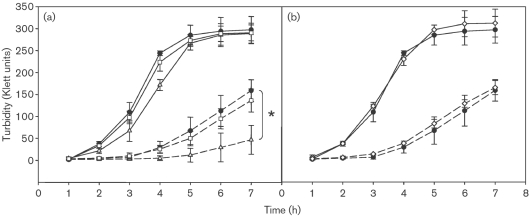
Sensitivity of an *nsrR* mutant to paraquat. Growth was measured in the absence (solid lines) or presence (dashed lines) of 500 μM paraquat. (a) Comparison of wild-type (•) with *nsrR* (▵) and *nsrR hmp* (□) mutants; (b) comparison of wild-type (•) with *hmp* mutant (◊). Data are represented as means±sd of at least three independent experiments. *, *P*<0.05 at the 7 h data point using Student’s *t*-test.

**Fig. 7. f7:**
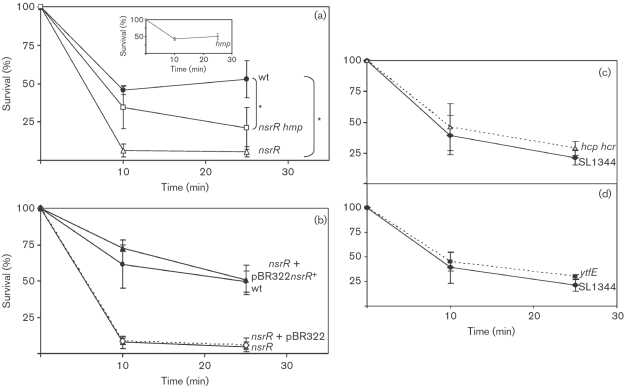
Percentage survival of cells after treatment with 5 mM H_2_O_2_. (a) Data are shown for the wild-type strain (•), the *nsrR* mutant (▵) and the *nsrR hmp* double mutant (□). (a) Inset, sensitivity of *hmp* mutant to peroxide stress. (b) Comparison of wild-type (•), *nsrR* mutant (▵), *nsrR* carrying pBR322 (dashed line) and *nsrR* carrying complementing plasmid (▴). (c, d) Sensitivity of *hcp hcr* (▵) and *ytfE* (▪) mutants, respectively, to H_2_O_2_ (dashed lines), and comparison with isogenic wild-type, SL1344 (⧫). Data are represented as means±sd of at least three independent experiments. *, *P*<0.05 at the 7 h data point using Student’s *t*-test.

We hypothesized that the compromised survival of *nsrR* mutant bacteria in macrophages is due to intracellular oxidative stress resulting from excessive Hmp synthesis and its reduction of O_2_ to 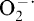 (Membrillo-Hernandez *et al.*, 1996; Wu *et al.*, 2004). This was tested *in vitro* using two different oxidative stress agents to study the response of wild-type, *nsrR*, *hmp* and *nsrR hmp* strains. Paraquat produces 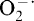 and is toxic to bacteria *in vitro* (Liochev & Fridovich, 1993; Halliwell & Gutteridge, 1999). In the absence of paraquat, all three strains grew to similar final densities although growth of the *nsrR* mutant, but not the double mutant, was significantly slower during most of the growth curve (Fig. 6a[Fig f6]). This is consistent with an inhibitory effect of excess Hmp synthesis in the absence of NO. Growth of the *nsrR* mutant in the presence of 200 μM paraquat showed some deficiency when compared to wild-type (data not shown). However, in the presence of 500 μM paraquat, growth of the *nsrR* mutant was almost completely inhibited (Fig. 6a[Fig f6]), whereas the *nsrR hmp* strain (Fig. 6a[Fig f6]) had growth characteristics similar to the wild-type, as did the *hmp* mutant (Fig. 6b[Fig f6]). These results demonstrate that Hmp, but not other products of the NsrR regulon, exacerbate paraquat sensitivity. The data also suggest that the poor survival of the *nsrR hmp* mutant in macrophages (Fig. 4c[Fig f4]) is due not to oxidative stress, but to the inability of the mutant to deal with nitrosative stress, as in the *hmp* mutant.


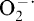
 spontaneously dismutates in solution to give peroxide (Halliwell & Gutteridge, 1999; Imlay, 2002). We therefore also performed sensitivity tests to H_2_O_2_. Strains were grown for 3 h before addition of 5 mM H_2_O_2_. Culture samples were taken immediately prior to the addition and then at 10 and 25 min following the addition. Viable counts were compared to the counts immediately prior to the stress (Fig. 7). Approximately 50 % of wild-type cells survived following 10 and 25 min exposure to H_2_O_2_ (Fig. 7a[Fig f7]). The *hmp* strain gave a profile almost identical to that of the wild-type (Fig. 7a[Fig f7] inset). The most severely affected strain was the *nsrR* mutant, with less than 10 % of cells surviving at 10 and 25 min after H_2_O_2_ treatment (Fig. 7a[Fig f7]). To confirm that the H_2_O_2_ sensitivity was a trait of the *nsrR* mutation and therefore overexpression of *hmp*, *nsrR* mutants carrying pBR322*nsrR*^+^ or pBR322 were also tested (Fig. 7b[Fig f7]). The *nsrR* mutant carrying pBR322 gave a killing profile identical to the *nsrR* mutation whereas the *nsrR* mutant carrying pBR322*nsrR*^+^ behaved like the wild-type. However, the *nsrR hmp* mutant also exhibited exacerbated H_2_O_2_ sensitivity, compared to wild-type, particularly after 25 min (Fig. 7a[Fig f7]). The reason for this result is unknown, although it seems possible that overexpression of a member of the NsrR regulon, other than Hmp, contributes to sensitivity to H_2_O_2_. Interestingly, recent studies in *E. coli* have shown that mutants in *hcp* are more sensitive to H_2_O_2_ (Almeida *et al.*, 2006), implicating Hcp in the oxidative stress response; thus overexpression of *hcp* is unlikely to increase oxidative stress sensitivity of the *nsrR hmp* mutant. The *Salmonella hcp hcr* and *ytfE* mutants were not increased in their sensitivity to H_2_O_2_ in these experiments (Fig. 7c, d[Fig f7]), indicating possible differences in the properties of Hcp in *E. coli* and *Salmonella*.

## DISCUSSION

This study has confirmed that NsrR is a major regulator of *hmp* in *S. typhimurium* both aerobically and anaerobically (Fig. 1b, c[Fig f1]), and mutation in *nsrR* protects against the effects of GSNO *in vitro*. This protection is shown to be a direct consequence of overexpression of *hmp*, as removal of *hmp* in an *nsrR* mutant abrogates the extra protection and leads to a growth pattern, in the presence of GSNO, that is similar to that of a single *hmp* mutant (Fig. 2). The absence of NsrR, with consequent overexpression of Hmp, also protects respiration from inhibition by NO (Fig. 3). However, in contrast to the growth effects, we show that this is due, not only to overexpression of *hmp*, but also to the overexpression of other gene(s) since, in the *nsrR hmp* double mutant, respiration recovers in a manner similar to the wild-type strain. In an *hmp* single mutant, respiration does not recover over the time period of the experiment, presumably due to the failure of Hmp to detoxify NO. Thus, no other members of the NsrR regulon can protect growth from the adverse effects of nitrosative stress (GSNO) but one or more of the other members of the NsrR regulon (*hcp*, *hcr*, *ytfE*, *ygbA* or other unidentified gene) are involved in protection of the oxidative electron-transport chain from inhibition by NO, at least in the absence of functional *hmp*. *tehA* and *tehB* expression in the *S. typhimurium nsrR* mutant was not increased, whereas in *E. coli tehA* was shown to be weakly regulated by *nsrR* (Bodenmiller & Spiro, 2006). The *tehA* and *tehB* gene products are involved in resistance to tellurite and other toxic compounds (Turner *et al.*, 1997) but roles for these genes in nitrosative stress resistance are unknown.

It has been suggested that *ytfE* may have a role in [Fe–S] cluster biogenesis in *E. coli* (Justino *et al.*, 2006). In *S. typhimurium*, *ytfE* has been identified as having a promoter that is maximally induced by 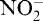 in a pH-dependent manner (Kim *et al.*, 2003). Mutants in *ytfE* are less readily cleared by mice in low-dose infection, the mechanism of which remains unknown (Kim *et al.*, 2003). Hcp contains two [Fe–S] clusters of unusual redox properties, and Hcr has sequence similarity to flavin-containing and [Fe–S]-containing class 1 NADH oxidoreductases and has been shown to reduce Hcp in the presence of NADH (van den Berg *et al.*, 2000). Like *ytfE*, the *hcp hcr* genes have also been shown to be optimally expressed in 1 mM 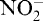 at pH 5, induced in NO-producing macrophages and less readily cleared by mice in low-dose infection (Kim *et al.*, 2003). Recently, the *E. coli* Hcp has been implicated in the oxidative stress response (Almeida *et al.*, 2006). The *ygbA* gene has no known function, although it has been shown to be regulated by *nsrR* in *E. coli* (Bodenmiller & Spiro, 2006).

Despite an *nsrR* mutant being hyper-resistant to GSNO and NO *in vitro* (Figs 2 and 3), we show that tight regulation of *hmp* expression is of paramount importance for survival in murine macrophages (Fig. 4), where bacteria will experience a range of stresses. Preliminary experiments assessing the survival of *hmp* and *fur* mutants in naïve macrophages suggested that the nitrosative stress response in these macrophages is not strong enough to attenuate survival of the *hmp* mutant (Fig. 4a[Fig f4]) but other stresses, particularly levels of radical and oxidizing species, have deleterious effects on the intracellular survival of a mutant in the global regulator, Fur (Fig. 4a[Fig f4]). Previous work looking at the intracellular survival of a *Salmonella* SL1344 *fur* mutant in macrophages demonstrated that this strain was not severely attenuated in intracellular survival (Garcia-del Portillo *et al.*, 1993). Later experiments by Wilmes-Riesenberg *et al.* (1996) also showed that the SL1344 *fur* mutant was not attenuated in its survival within J774.2 macrophages but that the degree to which a *fur* mutation affects virulence depends on the background strain of *Salmonella*, with the SL1344 *fur* mutant showing only a small increase in LD_50_ in mouse infection. These results could indicate why the 14028s *fur* strain used in this study shows an attenuation in J774.2 cells not seen before.

IFN-*γ* was used to enhance the antimicrobial nitrosative response. IFN-*γ* is the major macrophage-activating cytokine (Unanue, 1993). The resulting activation of iNOS is illustrated by the reduced survival of the *hmp* mutant (Fig. 4b[Fig f4]), which was significantly lower than wild-type in activated cells. The fall in *hmp* bacterial counts demonstrates the impaired ability of this strain to withstand nitrosative stress due to lack of the NO-detoxifying globin. 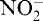 and 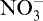 accumulation in tissue culture supernatants of infected cells confirms the enhanced activity of iNOS in the presence of IFN-*γ* (Fig. 5). *Salmonella* are known to interfere with the localization of iNOS (Chakravortty *et al.*, 2002), which may be enough to defend against RNS damage in the non-stimulated J774.2 but, in activated cells, where greater amounts of RNS are produced, Hmp appears to be required. This is in agreement with Bang *et al.* (2006), who showed a role for Hmp in acute and chronic mouse infection models and also presented data showing that a *hmp* mutant is not attenuated in infection of mice fed with the iNOS inhibitor L-NIL. Generally, RNS production by macrophages is thought to cause bacterial cell death via (in)activation of enzymes, ion channels and transcription factors, and mutation of DNA by strand breakage (Szabo *et al.*, 1996). However, a mechanism involving the IFN-*γ*-stimulated production of NO causing an inhibition of *Salmonella* SPI-2 effector expression has been reported, triggering the *Salmonella-*containing vacuole to interact more efficiently with compartments of the lysosomal/endosomal system, resulting in increased effectiveness of *Salmonella* killing by macrophages (McCollister *et al.*, 2005).

**Fig. 8. f8:**
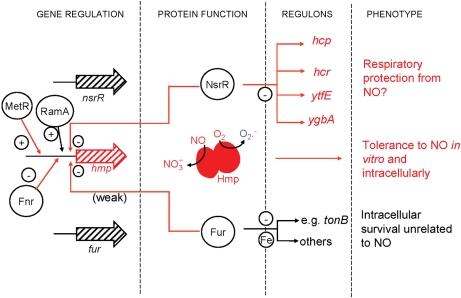
Contributions of NsrR, Hmp and Fur to NO tolerance and intracellular survival in *Salmonella*. Entities (genes, proteins, regulatory events, metabolic products) that increase on nitrosative stress are shown in red. Positive (+) and negative (−) gene regulation patterns refer to the effects of regulators in the *absence* of NO. Superoxide production from Hmp, which decreases with NO, is shown in blue. Transcription of *hmp* is positively regulated by MetR (in response to NO^+^-mediated homocysteine deprivation) and RamA (on exposure to superoxide), but negatively regulated by Fnr, Fur (weakly) and NsrR (strongly). Hmp catalyses the aerobic conversion of NO to nitrate, but (predominantly at the flavin domain) also reduces oxygen to superoxide in the absence of NO. In the presence of NO, repression by NsrR of *hcp*,* hcr*,* ytfE* and *ygbA* is lifted, and repression by Fur of at least 30 (Ratledge & Dover, 2000) genes is also lifted. At the right are shown the roles revealed in the present work of Hmp and the NsrR and Fur regulon components.

With enhanced expression of *hmp* and other genes in the NsrR regulon, it was predicted that the *nsrR* mutant might have an advantage over wild-type *S. typhimurium* in survival within IFN-*γ*-activated macrophages. However, our results suggest quite the opposite, both the *nsrR* and the *nsrR hmp* mutants being attenuated in IFN-*γ*-activated macrophages (Fig. 4c[Fig f4]). Furthermore, *in vitro* work demonstrated that the *nsrR* mutant is hyper-sensitive to both paraquat (Fig. 6a[Fig f6]) and H_2_O_2_ and that the *nsrR hmp* mutant is sensitive to H_2_O_2_ (Fig. 7). We have previously demonstrated that the presence of haem and FAD in Hmp not only provides a facile electron transfer from NAD(P)H to O_2_ and NO in the active site where 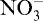 formation occurs, but also renders the protein susceptible to participation in additional redox chemistry. Specifically, reduction of oxygen to 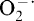 was first proposed by Orii *et al.* (1992), then demonstrated experimentally (Membrillo-Hernandez *et al.*, 1996; Wu *et al.*, 2004). Hmp acts as a reductase of broad specificity, reducing oxygen, cytochrome *c* and Fe(III) hydroxamate K, apparently without the involvement of the haem, since cytochrome *c* reduction can be demonstrated in the presence of CO (Poole *et al.*, 1997). Recent work in *S. typhimurium* (Bang *et al.*, 2006) has confirmed that the FAD-binding domain of Hmp mediates hyper-susceptibility to oxidative stress. Although these authors suggest specifically the reduction of Fe(III) to Fe(II) as demonstrated before (Poole *et al.*, 1997), and subsequent Fenton reaction chemistry (Woodmansee & Imlay, 2003), the reductive abilities of Hmp might facilitate numerous other reactions. Fig. 8 illustrates the network of interactions centring on Hmp. A large corpus of biochemical and physiological data supports the concept that Hmp possesses critical O_2_-dependent NO-detoxifying activity yet also has the potential for generating ROS in the absence of NO. A multitude of layers of transcriptional regulation provide the fine-tuning of Hmp levels necessary. Furthermore, additional modes of protection from nitrosative stress are clearly indicated by the current results from the *nsrR hmp* construct. Further work is required to assess the role of other NsrR regulon members.

It is notable that, within the intracellular environment, the *nsrR* strain appears unable to withstand the oxidative stress emanating from both macrophage and Hmp, despite the possession by *S. typhimurium* of several antioxidant enzymes including four SODs, namely two periplasmic Cu, Zn-SODs (SodC_I_ and SodC_II_) (Fang *et al.*, 1999), a Mn-SOD (SodA) (Tsolis *et al.*, 1995) and an Fe-SOD (SodB). SODs dismutate 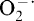 to H_2_O_2_, which can then be disproportionated into H_2_O and O_2_ by catalases. *S. typhimurium* also possesses two catalases, encoded by *katG* and *katE*. KatG and KatE are often referred to as HPI and HPII, respectively (for a review see Storz & Imlay, 1999). The results presented here suggest that these mechanisms of protection are overwhelmed by excess 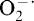 production in the *nsrR* mutant when Hmp is overexpressed. *In vitro*, the *nsrR hmp* double mutant is not affected by paraquat at the concentration used here but is affected by H_2_O_2_, although to a lesser degree than an *nsrR* mutation alone. This suggests that overexpression of a member(s) of the NsrR regulon other than *hmp* also enhances sensitivity to H_2_O_2_.

## Figures and Tables

**Table 1. t1:** Bacterial strains, phage and plasmids used in this study

**Strain, phage or plasmid**	**Genotype**	**Source/reference**
***S. typhimurium***		
ATCC 14028s	Wild-type	Crawford & Goldberg (1998b)
ATCC 14028s *hmp*	*hmp* : : *kan^r^*	Crawford & Goldberg (1998b)
ATCC 14028s *fur*	*fur* : : *kan^r^*	Kehres *et al.* (2002)
ATCC 14028s *nsrR*	*nsrR* : : *cat*	This study
ATCC 14028s *nsrR hmp*	*nsrR* : : *cat*, *hmp* : : *kan^r^*	This study
SL1344	*Xyl hisG rpsL*	
SL1344 *hcp hcr* (*nipAB*)	SL1344 Δ*hcp hcr* : : *kan^r^*	Kim *et al.* (2003)
SL1344 *ytfE* (*nipC*)	SL1344 Δ*ytfE* : : *kan^r^*	Kim *et al.* (2003)
SL1344 *nsrR hcp hcr*	SL1344 *nsrR* : : *cat*, Δ*hcp hcr* : : *kan^r^*	This study
SL1344 *nsrR ytfE*	SL1344 *nsrR* : : *cat*, Δ*ytfE* : : *kan^r^*	This study
**Bacteriophage**		
P22		Lab stock
**Plasmids**		
pTP223	*λred*, Tet^r^	J. Green, University of Sheffield
		Poteete & Fenton (1984)
pBR322	Ap^r^ Tet^r^	Lab stock
pBR322*nsrR*^+^	Ap^r^	This study

## Abbreviations

- Cm, chloramphenicol
- FCS, fetal calf serum
- GSNO, *S*-nitrosoglutathione
- IFN-*γ*, interferon-*γ*
- iNOS, inducible nitric oxide synthase
- NOS, nitric oxide synthase
- Phox, NAD(P)H oxidase
- qRT-PCR, quantitative real-time PCR
- RNS, reactive nitrogen species
- ROS, reactive oxygen species
- RT-PCR, reverse transcriptase PCR
- SOD, superoxide dismutase
